# New York County Veterinary Medical Association

**Published:** 1896-06

**Authors:** Robert W. Ellis

**Affiliations:** Secretary


### NEW YORK COUNTY VETERINARY MEDICAL ASSOCIATION.

THE regular meeting of this Association was called to order at the Academy of Medicine, on Tuesday evening, May 5th, by President Dr. Huidekoper, at 8.45 o'clock. On roll-call the following members responded: Drs. J. S. Cattanach, J. S. Cattanach, Jr., Delaney, Ellis, Ferster, Giffen, Gill, Huidekoper, Hanson, MacKellar, Meher, O'Shea, Robertson, Sherwood, and Turner (15). The minutes of the previous meeting were read and approved.

The Board of Censors having no business to report, and there being no papers, the time was given to discussion, which was opened by Dr. Robertson, who asked whether the Board of Health had a right to take possession of and destroy animals that show a reaction to the use of mallein, without any other outward signs of glanders? A general discussion followed on that point and on the symptoms of glanders in its various stages, and, finally, the following resolution was offered: "That the chair appoint a committee of three to communicate with the Boards of Health of the city of New York and the State of New York, and to investigate the value given by law to the inoculation of glanders by mallein, and the extent of authority of the Boards of Health in quarantining and condemning suspicious cases of glanders."

The chair thereupon appointed the following committee: James L. Robertson (Chairman), H. D. Gill, and Robert W. Ellis (Recorder).

Dr. Gill gave a few reports on the use of antitoxin in tetanus, with negative results. Dr. Hanson reported a case in which the symptoms became aggravated immediately after the injection of the antitoxin, which did not abate until relieved by death, which soon followed. Board of Censors, Dr. O'Shea, chairman, reported that our long-hoped-for Jury Bill failed to pass the Senate. He then read a list of names of men registered in New York County since October 1, 1875, followed by a report on the investigation made by the committee, on cards that were handed in at the last meeting, to the effect that none of the men to whom their attention had been called were registered, but that they had so far failed to get an interview with them. Moved and seconded that the report be accepted; carried.

Next in the order of business was the reading by the Secretary of the resignation of J. H. Ferster, V.S. The chair then asked the meeting their pleasure in the matter. Moved by Dr. Hanson and seconded by Dr. O'Shea that the matter be laid over until the next meeting. Amendment by Dr. Gill that it be acted upon at the present meeting. Vote on amendment lost. Vote on the motion carried.

Moved and seconded that a committee of three be appointed by the chair to draft resolutions for alteration of that section of the by-laws covering the night of meeting; carried. The following committee was appointed: H. D. Hanson (Chairman), J. S. Cattanach, and T. Delaney.

Moved and seconded that the Secretary be authorized to obtain a bill from the Chairman of the Judiciary Committee for expenses of that committee; carried. Dr. O'Shea then notified the chair that a committee from the Horseshoers' Association was outside, with the request that the V.M.A.N.Y.C. indorse their candidate for master horseshoer on the Horseshoers' Examining Board, Mr. O'Neil, and that the V.M.A.N.Y.C. appoint one of its members as candidate for Veterinary Examiner on said board.

Moved and seconded that the chair appoint a committee of three to recommend the name of Mr. O'Neil to the Governor as a member of the Horseshoers' Examining Board, and with the power to appoint a veterinarian. The following committee was appointed: Thomas Giffen (Chairman), H. D. Hanson, and F. W. Turner.

Moved, and seconded that the members of the V.M.A.N.Y.C. elect a veterinarian to the examining board at the present meeting; carried. Dr. Gill was unanimously elected.

Moved and seconded that the meeting adjourn; carried.

ROBERT W. ELLIS, D.V.S.,
Secretary.

